# Zimbabwean civil society survival in the post-coup environment: a political settlements analysis

**DOI:** 10.1057/s41309-023-00186-3

**Published:** 2023-03-23

**Authors:** Chipo Dendere, Shingirai Taodzera

**Affiliations:** 1 Department of Africana Studies, Wellesley College, Wellesley, USA; 2 Department of African and African American Studies, University of California, Davis, USA

**Keywords:** Interest groups, Zimbabwean democracy, Political settlements, Post-coup politics, Civil society, Authoritarianism

## Abstract

The political environment in post-independence Zimbabwe has been very hostile for interest groups, particularly since 2000 when civil society actions and opposition political activity formidably challenged the power and dominance of the ZANU PF party. However, the 2017 ouster of Robert Mugabe gave rise to renewed hope for political change. Using the political settlements theoretical framework, and data obtained from interviews with Zimbabwean civil society leaders, studies of legal documents, and government pronouncements, we analyze the changes in the relationship between the state and civil society groups during President Emmerson Mnangagwa’s regime. We find that, while civil society faced restrictions during Mugabe's tenure, their existence is now under much more scrutiny under Mnangagwa’s militarized government. This is because authoritarian regimes are more likely to increase the suppression of citizen voices when faced with growing citizen engagement.

## Introduction

Democracy has had a good trajectory across the African continent. In the last thirty years, most countries have held successful and uninterrupted elections, and there has been a turnover in leadership and sustained peace in most countries (Cheeseman 2015). Much of this progress can be credited to the sustained growth and independence of interest groups and civil society. For example, Zimbabwe has over 35,000 formally registered (and hundreds more that operate informally) civil society organizations that remain crucial in maintaining space- albeit limited- for democratic discourse. Zimbabwean politics cannot be understood, nor can it exist without civil society (Chipato et al. 2020). These diverse organizations include churches, community groups of women and youth, groups representing minorities, especially in the disabled communities, non-governmental organizations, and ordinary citizens working informally to solve community and political issues.

The structure of Zimbabwe's civil society is beholden to the regime's whims forcing CSOs to constantly change their strategies in response to the prevailing political climate. In the early nineteen twenties, the earliest formalized CSOs were created to cater to the needs of rural women (Chipato et al. 2020). In the fifties, local groups formed to lobby the white colonial settler government for black people's issues. Eventually, many of these groups would unite under the banner of the anti-colonial movements. Between independence in 1980 and 1999, most civil society activities were focused on development issues, although a small minority continued to work on civic education. Civil society groups were also folded into trade unions and labor activism. However, the HIV pandemic again shifted the focus on CSOs, with many organizations pivoting to work on AIDS-related issues. The shifts in CSO focus until then had primarily been influenced by need and available funding. In the post-2000 era, as political and economic conditions deteriorated, opposition parties' strength increased, and civil society organizations became more preoccupied with political issues and human rights. Because CSOs working on political rights were naturally aligned with the opposition movement, the government became highly hostile toward civil society leading to the unjust imprisonment of hundreds of political and non-governmental actors.

Civil Society enjoyed a brief period of freedom in the immediate period shortly after the 2017 coup as President Mnangagwa sought to dissociate his regime from that of his predecessor. That moment has since gone, and democracy again faces further decline. We begin our analysis from the 2018 election period. Our motivating question is twofold—first, we unpack why Mnangagwa’s regime is regressing on its post-coup promise to foster democracy. Next, we are interested in the strategies used to sabotage civil society and how does civil society respond to these challenges? We answer this question using data from primary and secondary sources. We conducted ten interviews with leaders of Zimbabwe-based civil organizations over the phone as the work was completed during the early part of the COVID-19 pandemic. In the Zimbabwean context and other authoritarian regimes, it is not easy to convince people to speak openly, and as such, the data will be anonymized. We also use data from secondary sources, including the analyses of legal documents and government pronouncements.

Using the Political Settlements analytical framework, we show that although the authoritarian ZANU PF regime defaults to suppressing citizen voices using the armed forces and politics of patronage, Mnangagwa’s ZANU PF regime is caught in a trap between maintaining the existence of a semblance of democracy in Zimbabwe, which includes having active interest groups and civil society and constraining the activities of the Third Estate, which are, in essence, a threat to its grip on power. We argue that authoritarian regimes such as the ZANU PF government suppress interest groups when the level of accountability that an open and unrestricted civil society would engender threatens its security of political tenure. This explains why Mugabe and Mnangagwa’s regimes adopted similarly aggressive approaches to civil society actors. Since seizing power in 2017, the ruling party has gradually shrunk the little political space that opened following the 2017 coup by simultaneously using and co-opting some civil society groups and using heavy-handed methods.

## Zimbabwe after Mugabe: background

In November 2017, officers from the Zimbabwe National Army (ZNA) ousted former president Robert Mugabe, who had been in office since Zimbabwe attained independence from colonialism in 1980 from office in a guardian coup. A guardian coup occurs when the military promises minimal political intervention, and their involvement will end as soon as there is a qualified civilian leadership (Dendere 2017). Unlike coups elsewhere on the continent, various civil society organizations (CSO) and the leading opposition party, the Movement for Democratic Change (MDC), were very actively involved in the coup and called for Mugabe to step down. There was a general expectation that the political demise of Robert Mugabe would lead to a more politically open Zimbabwe. There was even some hope that his successor, Emmerson Mnangagwa, whom the military-appointed, might lead to democratic reform like Ghana’s Jerry Rawlings, who came in via a coup and successfully transitioned toward democracy (Rwodzi 2019).

In the first months of his presidency, Mnangagwa’s regime appeared to be on a path toward democracy. During the post-coup election period between November 2017 and May 2018, the opposition campaigned with more freedom than they had previously. Leaders from various civil society organizations interviewed for this paper also reported that their organizations could do their work with very little government interference. Although most international observers could not enter Zimbabwe to observe the elections, regional and local groups could freely mobilize voter registration and get-out-the-vote campaigns. Organizations working on food aid distribution had unrestricted access to citizens, especially in peri-urban and rural areas. Under the Mugabe regime, it was not always easy to access rural areas, and in rare cases, food distribution was partisan (Mwonzora & Mandikwaza 2019). One CSO leader said, “it was refreshing to get people the things they needed without fear” (Lovemore 2021). Local leaders in diverse communities shared her sentiments.

The 2018 election voting day was, for the most part, peaceful. Most people who wanted to vote could do so with minimal difficulty or interference from the police or other political actors. However, the peace was short-lived. A day after the election, on August 1, 2018, a group of youth, some affiliated with opposition political parties and others motivated to protect their democratic vote, took to the streets to protest the delayed release of election results. When individuals protest in authoritarian states like Zimbabwe, there is a general expectation that the police might respond with a rather heavy-handed approach that mainly involves teargas or water cannons. However, most protestors and observers did not expect the government to respond by sending heavily armed soldiers to react to unarmed civilians. The army killed six bystanders in broad daylight, and many others were injured. After months of lobbying by civil society organizations, the government commissioned an inquiry committee. However, no officials have been held accountable since then. The post-election violence by the military in August 2018 marked a shift in the Mnangagwa regime’s public interaction with media, civil society, the international community, and citizens. Although the military had always been involved in politics, this was the first time that armed soldiers opened fire against citizens. Whatever goodwill had been built following the Mugabe ouster was eroded. The message was clear: organizing, mobilizing, speaking out, and protesting would not be tolerated. The gun would have the final say in politics.

To examine why the ZANU PF regime typically suppresses civil society, we situate our analysis of Zimbabwe’s political system in the political settlements framework. This involves providing a discussion of the party’s history, influential actors within its ruling coalition, sources of party power within the Zimbabwean case, consequences for governance in general, and the party’s suppression of interest groups. We also outline a selection of laws and regulations used by the post-coup ZANU PF government to suppress citizen engagement and elevate the participation of a small subset of organizations that promote the party’s agenda and propaganda. We then examine survival strategies used by civil society as a spectrum that includes formal, semi-formal, and informal organizational configurations. The article contributes to understanding how interest groups exist and operate within authoritarian hybrid regimes and the implications of authoritarian populism on organized civil society through an empirical case in Africa.

## Explaining Mnangagwa’s ZANU PF regime’s stance: a political settlements lens

“Political settlement” refers to the distribution of power between influential groups in a particular country that is a product of the struggle between these groups and influences the distribution of power (Khan 2018). Political settlements also arise out of the deals and compromise that groups make at elite and non-elite levels (Khan 2018). The political settlements approach views competition and the struggle for political power and material resources as the primary factor in understanding the dynamics of human interaction (Gray 2019). Thus, a ‘settlement’ denotes a balance of power that can be discerned over a relatively long period, spanning a few years. (Khan 2018). This balance of power influences political outcomes, such as the institutions and policies that define and regulate life and other political events. It also affects the existence and function of other political actors operating within the established political settlement.

Political settlements are subject to ongoing change, resulting, for instance, from political events, such as elections or a coup. This leads to other configurations of power that have corresponding effects on a country’s institutions (Gray 2019). The patterns of political competition resulting from either elite or non-elite action alter the configurations of power in favor of specific groups, coalitions, or regimes (Gray 2019). Bargains and deal-making between elite actors and non-elite supporters also shift the status quo, establishing political institutions or outcomes that mutually benefit them (Di John and Putzel 2009). This power shift becomes a ‘settlement’ when it is acknowledged and accepted by other competing actors, giving way to stability and institutional developments.

Political settlements can be classified as inclusive, exclusive, and transitional. Inclusive settlements, commonly associated with developed western societies, entail a political-administrative system whose institutions promote efficiency and long-term institutional stability and where independent government bureaucracies largely control political and distributive power instead of informal patronage networks (Khan 2010). Exclusive settlements arise when political and institutional power lies in a small coalition of actors that exploits public institutions for their partial gains at the expense of most of the population (Kelsall 2016). This is often the case in developing nations with relatively underdeveloped political institutions, whereby public goods are distributed based on patronage politics instead of impartial institutions of governance. Transitional settlements describe a scenario where the redistribution of power is underway. Transitions can be recurring political events, such as elections, a political coup, or any other challenge to the incumbent coalition’s power and hegemony, potentially resulting in a reconfiguration of the balance of power (Kelsall 2016).

The analytical pillars of the Political Settlements framework are historical dynamics, actors and their interests, sources of power, and institutional outcomes (Behuria et al. 2017; Khan 2018; Gray 2019). The historical analysis provides vital background information that explains observable political dynamics. In Africa, for instance, histories of colonial rule, the Cold War, and Structural Adjustment Programs (SAPs) considerably shaped the nature and function of political institutions that define various aspects of life in the present day. Secondly, the identity of specific political actors, who can be individuals or groups at the elite and non-elite levels, and their interests is a key component of political settlements because actors’ identities and interests are often intertwined, which explains their collaboration and contestation patterns. For instance, some post-colonial African countries have had ethnically patterned political mobilization and conflict, such as the Gukurahundi genocide in Zimbabwe between 1982 and 1987, the genocide in Rwanda in 1994, and more recently, the civil war in Ethiopia. Indeed, ethnic identities often underlie patronage networks through which holders of political office distribute public goods or other forms of material gain (Berman 1998).

Power is another fundamental feature of the political settlements approach, particularly its relational nature. It is defined as an actor’s capacity to impose their will over others or influence their choices or actions directly or indirectly (Dahl 1957; Weber 1965). The sources, nature, and extent of actors’ power influence competing actors’ reactions, shape the patterns of competition or collaboration, and subsequently impact outcomes. In other words, power is the ‘currency’ of politics. The fourth analytical component of the political settlements approach is that of political institutions. Unlike institutional analysis, which focuses on the extent to which the nature and quality of public institutions and governance explain political outcomes, political settlements view the nature and function of formal institutions and policy outcomes as a result or a reflection of underlying informal power dynamics (Khan 2018). Powerful coalitions establish and support institutions that guarantee their interests and vice versa.

Several scholars have employed this approach to analyze various issues in African studies. For example, Jonathan DiJohn, James Putzel, and Amy Poteete applied the framework to analyze Botswana’s political system and management of the diamond sector, finding that its political stability is not necessarily based on institutions alone but the political negotiations and bargaining between powerful domestic and external actors: the ruling Botswana Democratic Party and its leadership, traditional chiefs, cattle ranchers, De Beers Diamond Corporation, and the British government (Di John and Putzel 2009; Poteete 2009). Taodzera (2020) applies the political settlements approach to disprove speculation of an impending ‘resource curse’ arising in Uganda, prescribing a comprehensive, multi-pronged analysis of the governance of the oil sector instead of employing the conventional analyses that privilege institutions of Weberian standards, mostly present in ‘First World’ countries (Taodzera 2020). Abdulai and Hickey also use the approach to study Ghana’s government’s spending patterns in the education sector, finding that the level of control that regional elites wielded in central government corresponded to the degree of funding allocated to their home regions, which revealed the influence of underlying political settlements on national formal education policy outputs (Abdulai and Hickey 2016).

These brief examples demonstrate the Political Settlements approach’s utility in facilitating a comprehensive understanding of the nature of the ZANU PF ruling coalition and explain its suppression and heavy-handed response to interest group action in Zimbabwe, particularly under President Mnangagwa’s government. In the next section, we apply its analytical perspective to dissect the party’s sources of political power and how this influences its quasi-accommodative strategy on interest groups and civil society in general.

### A history of colonial rule and revolutionary struggle

The roots of the ZANU PF regime’s authoritarian nature lie in the history of militarized anti-colonial activism. Having been a settler colony, Zimbabwe’s transition to independence entailed a protracted struggle between various armed and unarmed groups, notably the settler colonial government of Ian D. Smith (Evans 2007). Months of targeted military attacks from ZANU’s army forced the Smith regime to negotiate with the black politicians at the Lancaster House peace talks in 1979. The Lancaster peace negotiations culminated in the 1980 elections that brought Mugabe into office. The majority of ZANU’s war-era leadership became part of the new democratic government, including the current President Mnangagwa and his deputy, General Constantino Chiwenga. Although it claims to be a democratic civilian-led party, ZANU PF is, at its core, still a hierarchical military movement inhospitable to internal and external dissent. Its history of militarization has had far-reaching effects on its present-day structure and culture (Southall 2013).

The party is beholden to a top-down hierarchical system as a formerly armed movement. The leadership is intolerant of dissent, regarding it as disloyalty and, at its worst, ‘selling out, resulting in ostracization or expulsion (Mlambo and Raftopoulos 2010). This is demonstrated in ZANU PF’s trajectory in the post-independence, whereby prominent ZANU PF leaders who criticized Mugabe were expelled from the party and wound up in political opposition (Ndlovu-Gatsheni 2008). Such (popular) leaders include the late Margaret Dongo, Eddison Zvobgo, and former finance minister Simba Makoni in more recent times. Therefore, ZANU PF’s culture of intolerance to dissent extends to interest groups and civil society organizations. Interest groups and civil society are typically regarded as ‘sellouts’ working at the behest of countries such as the USA and the United Kingdom, the former colonial power, to return the country to colonial subordination. ZANU PF’s intolerance of citizen-led interest groups partly has roots in its history as an armed movement with a rigid hierarchical structure and weaponization of liberation ideology to attack civil society groups and any other perceived threats to its hold on power.

### ZANU PF’s leadership and intertwined political and economic interests

We focus on the post-coup leadership of ZANU PF under President Emmerson Mnangagwa and his deputy, former army commander General Constantino Chiwenga. The two men pull the levers of power and have primarily been responsible for the onslaught of violence against interest groups and civil society activity in Zimbabwe. Mnangagwa participated in the liberation struggle as part of a crop of young guerilla fighters who received military training at the height of the anti-colonial movement in then Rhodesia under the banner of the Zimbabwe African National Liberation Army (ZANLA), the armed wing of the ZANU party (Tendi 2016). One of the defining features of Mnangagwa’s career is his close working and personal relationship with Mugabe, and he was long viewed as his successor. Mnangagwa had deep links with the military leadership, notably his close allyship with Chiwenga. This explained the army’s actions in neutralizing factions of the security sector loyal to Mugabe and installing Mnangagwa into office during the coup in 2017 (Cropley 2017). Mnangagwa has, for decades, been steeped in ZANU PF’s culture and leadership structures and has a well-documented authoritarian streak that has been manifest since he seized power.

Chiwenga, who never held political office until his appointment to the vice presidency in 2017, is also a former combatant and rose through the ranks to become the ZANLA Deputy Political Commissar^1^ in 1978 (Zulu 2017). At independence in 1980, he was incorporated into the newly established Zimbabwe National Army, rising to Lieutenant-General in 1994, then commander of the Zimbabwe Defense Forces in 2004 (Zulu 2017). He held this position until his retirement and appointment as vice president in 2017. However, he remains influential in the security services sector. He is chair of the Joint Operations Command, which brings together all the country’s security organs (army, air force, intelligence, police, and prison services.) Most importantly, Chiwenga coordinated the coup that brought Mnangagwa into power after his flight to exile in South Africa following his dismissal by Mugabe in 2017. Chiwenga is, in effect, the force behind the throne (Harvey and Alden 2020). As a career soldier since the liberation era, Chiwenga has been grounded in the hierarchical institutions and cultures of the army that are antithetical to the dialogic, interactive nature of democracy, of which interest groups are an essential component.

Although these two leaders have considerable influence over the party and government, it is essential to note that ZANU PF is not monolithic and homogenous at this juncture. Instead, it is a coalition of groups vested in the party’s continued power. At the elite level is the political leadership, from its top decision-making organs such as the Central Committee^2^ and Politburo^3^ to its provincial and district structures, chief security officers in the army, police, intelligence, and prisons, a faction of the war veterans, and militia groups such as Chipangano, which the party uses to attack and intimidate civil society and opposition party activists (Mutongwizo 2014). At the non-elite level, the regime has an extensive patronage network, which consists of the leaders mentioned above, their family members and acquaintances, supporters of the party, and businesspeople who act as both financiers of the party, and beneficiaries of its patronage through access to tenders and other lucrative business opportunities accessed through state entities.^4^

Thus, Mnangagwa, Chiwenga, and the rest of the ZANU PF top brass and members of the patronage cartel have a vested interest in maintaining hegemonic control over the country’s political system for political and economic reasons. The government is, in effect, the ZANU PF’s cartel’s means of access to material benefits and livelihoods. Several corruption scandals have revealed the deep web of linkages between top government officials and local and international beneficiaries.^5^ ZANU PF functionaries at various levels of the organization’s hierarchy depend on their political offices and the party’s hold over power for their livelihood. This was demonstrated by the degree to which several former leaders, expelled from the government after the November 2017 coup, fell on hard times soon after. Thus, the line between government business and private interests is blurred because of state capture and politics of patronage. Civil society organizations’ activities are, therefore, a threat to these intertwined objectives of the ZANU PF cartel: control of the state and access to economic benefits.

### Sources of power: state security apparatus, public institutions, and revolutionary propaganda

An analysis of the ruling of the ZANU PF regime’s primary sources of power demonstrates its inability to accommodate a vibrant and unconstrained civil society sector. Masunungure and Bratton (2011) show that the country’s military and security agencies, such as the Central Intelligence Organization (CIO) and the Zimbabwe Republic Police (ZRP), underwrite the regime’s power and extended stay in office (Masunungure 2011; Bratton 2014). Security agents maintain the status quo through direct attacks, threats, or violence against the political opposition and civil society activists who speak out against the government or challenge its power in any manner the regime deems threatening.

Secondly, the ZANU PF regime exploits state institutions, resulting in the conflation of ZANU PF as a party and the government of Zimbabwe. Government institutions have, since independence, operated in service of ZANU PF’s interests instead of being impartial distributors of public goods, mainly through sources of patronage and as tools for attacking political opponents. For instance, the party abuses state resources, such as public funds, vehicles, fuel, and state-owned media, mainly the Zimbabwe Broadcasting Corporation (ZBC), to run its political campaigns (Magaisa 2019; Ndakaripa 2020). It has a well-documented, profoundly entrenched culture of patronage and cronyism that includes irregularly awarding tenders to government officials and their cronies and outright theft of public funds through various strategies. Examples of large-scale corruption include the Willowvale car procurement scandal in 1988^6^ and, more recently, multiple corruption and fraud cases involving President Emmerson Mnangagwa’s close associate, Kudakwashe Tagwirei, and Vice President Constantino Chiwenga (Sharife and Anderson 2021).

Besides using the government as a cash cow, ZANU PF instrumentalizes the judiciary to attack political opponents (Magaisa 2019). The party has a well-documented record of employing intelligence agents and police officers to abduct and detain civil society and opposition activists for extended periods without trial (US Embassy in Zimbabwe 2017). Civil society activists and opposition leaders have been arraigned before the courts on trumped-up charges and subjected to lengthy, costly, and humiliating judicial processes (HRW Report 2008; FH Report: Zimbabwe 2021). Since 2018, ZANU PF has intensified its attacks on opposition activists from the leading opposition Citizens Coalition for Change (CCC) and numerous independent journalists(Muronzi 2022. Those who have suffered state brutality include a member of parliament who has been repeatedly denied bail and remains in prison after more than 200 days as of January 2023. Others include party spokesperson Fadzayi Mahere who is frequently called to court over frivolous charges. Activists from Matabeleland spent a night in jail after attending the Gukurahundi commemoration in December 2022, and have been repeatedly arrested and imprisoned for extended periods as part of the ZANU PF regime’s tactics to intimidate the leaders, their supporters, and allies). The regime employs “rule by law,” or judicial purposes, as a tool of political persecution to wear out opponents and instill fear in would-be critics. By and large, Zimbabwe’s judiciary is biased, and most rulings, especially in politically sensitive cases, go the way of the party (Magaisa 2020).

The ZANU PF regime’s third power source is ideological: the party’s revolutionary narrative and propaganda. ZANU PF, like South Africa’s African National Congress (ANC) and Namibia’s South West African People’s Organization (SWAPO), continually portrays its leaders as heroes of the anti-colonial, revolutionary activism and armed campaigns that delivered the country’s independence from colonial rule (Clapham 2012; Southall 2013). This message resonates with the party’s supporters, particularly those in rural areas. In this vein, the party deploys this ‘liberation ideology’ that any kind of opposition political organization and civil society activism against ZANU PF is ‘unpatriotic’ and a threat to the country’s independence—thus conflating its definition of patriotism with zealous support for ZANU PF (Ndlovu-Gatsheni 2011). As a result, opponents of the ruling party, both in the country’s opposition parties and critics in the civil society, are branded as ‘sellouts’ who endeavor to deliver the country back to the clutches of British colonialism and neo-imperial domination by other European countries and the USA.

This analysis of the ZANU PF party’s sources provides a nuanced explanation of the party’s hostility toward interest groups that do not openly support the regime. Since independence, the three wings of security agencies (army, police, and intelligence) have been the backbone of ZANU PF’s political power. This behavior and political strategy against opponents is rooted in the party’s history as an anti-colonial militia organization fighting for independence.

Impact on interest groups: Legislative authoritarianism and hostility to an open society in the post-Mugabe era.

The preceding analysis of Zimbabwe's political system provides an important background to the country’s civil society’s complicated relationship with the state in the post-independence era. While the government has openly received aid via NGO channels, CSOs have also been consistently met with suspicion and accused of being western-funded to destabilize the government (Dorman 2003; Kandemiiri 2013). ZANU PF has always been quick to silence civil society organizations that engaged in human rights-related work or criticized the government for human rights abuses, both by omission and commission. For example, during Mugabe’s time, Dr. Mashiri (not her real name) worked with a women’s trust organization in the early 2000s. She recalled heightened scrutiny on their work after the 2000 election, which saw ZANU PF losing its senate majority for the first time. Their organization has always been a trusted agency for advocating for victims of domestic violence and those living with HIV. Still, the government was suddenly concerned that their advocacy had become too political. Indeed, by 2004 ZANU PF minister of Public Service and Social Welfare, Paul Mangwana, was actively campaigning against civil society groups. In 2004, he was quoted saying the following about CSOSs:

Some NGOs and churches are causing too much confusion in the country because they are converting their humanitarian programs into politics … The government cannot allow that to happen, so we are saying they should go under scrutiny where we revise all modalities of their operations in the country.^7^

During Mr. Mangwana’s tenure, the government introduced the first bill geared toward giving the government more control over the work of NGOs and churches. A reverend affiliated with the Methodist church interviewed under anonymity for this paper recalls that starting in 2004, churches were asked to provide details about their membership. The government was concerned that churches were working with western journalists or harboring opposition activists on the run. By November 2004, the government had suspended the constitution to fast-track the NGO bill. Precedence for restrictive laws that prevented citizens from agitating for their rights had been set two years prior with the passing of the Access to Information and Protection of Privacy Act (AIPPA) and the Public Order and Security Act (POSA). These two legislative acts directly targeted media groups (Crisis Coalition 2004). The situation progressively worsened through the remainder of Mugabe’s term. However, CSOs remained resolute, and there was much active work on human rights and exposing government malpractice in various areas.

When the army began the process of removing Mugabe from office by force, pro-Mnangagwa activists in ZANU PF utilized the social CSO social network to mobilize people to support its coup against former president Mugabe and Mr. Mnangagwa’s post-coup government. Mr. Mutsvangwa, one of the ZANU PF spokespersons, told one of the researchers for this project that the party was aware that without CSO buy-in, they would not be able to get people onto the streets to protest against Mugabe. Some CSOs expected the new regime to be a better ally, given their role in Mugabe's ouster. Indeed, in the few months after Mugabe’s ouster, Mnangagwa’s language suggested that his government would be more open and that state-sponsored hostility to civil society would be a thing of the past. Mnangagwa promised jobs, peace, and fair elections in his inaugural speech. He indicated that the unfair jailing of activists was a thing of the past as he publicly embraced opposition leaders, including Morgan Tsvangirai.

However, in June 2021, the government threatened NGOs through rhetoric and legislation. Government spokesperson George Charamba said, “We cannot allow, internally, any organization which we register under our statutes to subvert the sovereignty and integrity of Zimbabwe” (Chidakwa 2021). In the same statement, the government repeated its narrative that many NGOs are working under a sole mandate for regime change and that “very few of them are helping to build schools, clinics, to give livelihoods to our people through irrigation schemes. All of them are employed to achieve regime change in Zimbabwe. We condemn it as they are calculated to undermine the orderly evolution of our political, economic, and judicial systems and must be condemned” (Chidakwa 2021).

Mnangagwa’s government also introduced restrictive laws to curtail civic space, a tactic frequently used during Mugabe’s tenure of office. The Private Voluntary Organizations (PVO) Amendment Bill to restrict civil society space, introduced in 2021, is a case in point. According to the government, the Private Voluntary Organizations Amendment Bill 2021 is being proposed “to comply with the Financial Action Taskforce (FATF) recommendations to Zimbabwe. Further, it has become necessary to streamline administrative procedures for private voluntary organizations to allow for efficient regulation and registration.” (GOV OF ZIMBABWE 2021). The government also claims that the bill will assist in preventing money laundering and bringing the country into compliance with international law. The bill is an amendment to the very restrictive Private Voluntary Acts (1996, 2002), and the proposed changes will give the executive more power to closely monitor civil society's activities and constant evaluation that their mission is complementary to that of the ruling party (Chifamba 2021b).

The bill is a draconian law designed to suffocate all non-ZANU PF civil society organizations. Only those whose activities strengthen the party’s power and burnish its image will survive. The PVO bill is part of the Mnangagwa regime’s hegemonic agenda to expand its dominance in every sphere of life, like the Chinese Communist Party with which it has close relations. In multiple interviews, Musa Kika, director of the Human Rights Forum, said that the government is increasingly aiming to reduce civil society activities, especially those that will push for a free and fair election environment in the 2023 electoral cycle. Activist-journalist Hopewell Chin’ono also argues that these laws will further entrench corruption. For instance, in 2020, he spent over 200 days in jail for publicizing that the then Minister of Health had embezzled over the US $60 million earmarked for COVID. To date, neither the former minister nor his top-ranking officials have spent time in jail. During our interview with Mr. Chin’ono, he said he did not think this bill was designed to deal with corruption. He said corruption is rooted in government structures and tenders and not within the CSO spaces, especially since the Mnangagwa regime has established a record of giving tenders to friends and allies who do not always have the requisite requirements for those deals.

Another draconian strategy to stifle civil society activity through monitoring and surveillance is President Mnangagwa’s creation of a new and largely unconstitutional role of ten Provincial Development Coordinators in August 2020 (Ncube 2020). Their job was described as assisting the Ministry of Local Affairs, another controversial ministry, with technical and administrative support. Efforts to lobby against the creation of this office by citizens and civil society failed. By December 2021, Mnangagwa announced that the Provincial Development Coordinators would be promoted and given even more powers that potentially curtail civil society activities. In June 2021, the Provincial Development Coordinator (PDC) of Harare, Tafadzwa Muguti, stated that all NGOs operating in the capital city, Harare, would now need to seek permission to use through his office beginning July 2021. Consistent with the new regime rhetoric, in his public address, Mr. Muguti emphasized that civil society and business entities seeking to work in Zimbabwe would be watched closely: “The first task is unbundling ministry duties to the province. I will be meeting captains of the industry to hear what they will be able to contribute to our provincial economic plan. Everything we do as Harare is under the spotlight. Anyone who flies into Zimbabwe meets us first.” (Munhende 2021).

This policy has no constitutional mandate, which violates freedom of speech and expression. The goal of these mandates is twofold, first, to criminalize the work of civil society organizations and, secondly, to trap them in a never-ending cycle of government red tape. The constant changes in government personnel due to intra-party fighting further complicates the civil society groups' ability to adhere to the regulations. The anticipated chaos started shortly after the announcement. In late July 2021, there was an attempt to ban all non-compliant organizations, which failed to take effect after civil society organizations filed a successful petition with the courts (Mabika 2021). Government officials had announced that out of the over 3000 CSOs working in Zimbabwe, only 40 were compliant. Ironically, their ban extended to the first lady’s controversial charity organization, Angel of Hope Foundation, partly explaining why the courts dismissed the ban announcement. Nevertheless, as expected, all other NGOs had spent their limited resources fighting another court battle they did not need to engage in (Human Rights Forum 2021). Mugabe’s government routinely used the same tools of repression against civil society, and now Mnangagwa is further refining the strategies.

### Developmental authoritarianism and Interest Groups in the post-Mugabe-era

Besides using the law to undermine civil society activity, Mnangagwa’s government has also employed co-option as part of his regime’s carrot-and-stick strategy. This strategy aims to diffuse the power of non-aligned interest groups and persons and centralize control within the state. To this end, the government has created interest groups like the Presidential Advisory Council, a 26-member Presidential Advisory Council (PAC) in 2019 consisting of eminent figures and experts from business, health, agriculture, civil society, faith-based organizations, and education a few months after the 2018 elections (The Herald Zimbabwe 2019). Mnangagwa indicated that the purpose for establishing the PAC was to receive expert and unbiased input in policy formulation, especially the government’s Vision 2030 agenda, from a group of knowledgeable and unbiased citizens (Staff Reporter_Zimbabwe Mail 2019). The council’s membership included formerly fierce opponents of ZANU PF in civil society and churches. This was consistent with Mnangagwa’s strategy to bring critics under his wing. As expected, public response was mixed; others criticized the bloated nature of the council and the costs involved, while others also lauded the president for a diverse and inclusive team of advisors.

Although the PAC is not the traditional type of interest group, its membership includes NGOs and civil society, so it is worth a brief discussion. For example, Kenneth Mtata, secretary of the Zimbabwe Council for Churches, has traditionally spoken out against ZANU PF. It is not surprising then that since the last election, church leaders have been more open in their support for the president and even, at one point, called for a suspension of elections, arguing that the nation needed an extended period of healing from politics (Anli 2019). In a country where over 90% of voters report a Christian affiliation, the church’s backdoor endorsement of ZANU PF is impactful. The formal church formations were also important allies for pro-democracy groups during the Mugabe era. However, they, too, have moved toward supporting the Mnangagwa regime. In interviews with various bishops, they indicated that their support for the new president was anchored in the optimistic belief that he would live up to his promises. Mnangagwa has also been successful at pitting younger church leaders who fit the bill of social media influencers who have millions of followers on social media against more older church leaders who are not easily bought off. Church leaders willing to work with the president have been rewarded with high-level ambassadorial posts. The goal of doing this is to cause fissures within various civil society structures.

In addition to targeting religious organizations, the Mnangagwa regime has also gone after the media. Early PAC members included media mogul Trevor Ncube who was forced into exile for over two decades during the Mugabe years for exposing state brutality. By including Mr. Ncube, Mnangagwa was signaling that he would work with independent media, an area previously closed off to ZANU PF. Mr. Ncube’s newspaper, The Independent, was among a dozen private media houses subjected to state-sponsored attacks, which included bombings since the early 2000s (Meldrum 2001). That Mr. Ncube accepted work with Mnangagwa sent a strong signal that he believed free and independent media had a chance under Mnangagwa. However, the militarized government of Mnangagwa cannot function alongside institutions of democracy. Although Mr. Ncube has yet to speak publicly about his experience within the PAC, he has indicated that he officially resigned from the organization, citing disagreements on democratic norms with the president (Chibamu 2022). Subsequently, the independent media has been left in chaos. Independent journalists continue to be harassed and jailed without course. The weakening of various interest groups strengthens the regime's strategies.

Mnangagwa’s government has also committed to changing the perceptions about ZANU PF's relationship with the business world and appointed several business executives to the PAC. This includes Natalie Jabangwe, EcoCash's partner company of ECONET, the largest company in Zimbabwe. This appointment was also critical because the founder and owner of ECONET, Mr. Strive Masiyiwa, has been in exile since 1999 following many attempts on his life by the government. This appointment signaled that Mnangagwa was extending an olive branch to many in the business world who felt unsafe under Mugabe. The strategy worked, at least for a little while. Although Masiyiwa did not physically return home, he used his public voice on social media to shore up support for Mnangagwa and provided much-needed financial support (Staff Writer, 2018). There was also a rush to return home by other business exiles such as Mutumwa Mawere (Guma 2010) and another media mogul, James Makamba (Staff Reporter 2017).

Although the initial excitement has since waned, and many exiles have returned to their more comfortable lives abroad, these appointments endeared Mnangagwa to civil society groups previously hostile to ZANU PF, mainly in the private media, religious organizations, local and foreign business interests, and non-governmental organizations. A PAC member who spoke on the condition of anonymity said they believed that Mnangagwa was interested in moving the country forward and utilizing their experiences from years of international development work and civil society to help the country. However, it also became clear that only some are interested in progress. They added that factionalism, corruption, and individual interests far outweigh the national agenda, and many PAC members feel frustrated. At this writing, two members of the PAC had officially quit, with others rumored to be on their way out. Mnangagwa appears relaxed about this as he continues forming other pro-regime interest groups.

In addition to forming the PAC and as part of his co-option strategy toward critics, Mnangagwa used his executive powers to make the Political Actors Dialogue (POLAD), a coalition of 2018 election losers minus Nelson Chamisa, leader of the Citizens Coalition for Change (CCC), the country’s largest opposition political party, and himself (Helliker and Chikozho Mazarire 2021). As with the PAC, POLAD brings together some of ZANU PF’s most vocal opponents, including Thokozani Khupe, Douglas Mwonzora, and Lovemore Madhuku. POLAD has justified its participation by arguing that this organization is part of a thriving civil society that sees political parties of different frameworks working together (Chifamba 2021a). Political parties merit mention here because they are essential to the civil society community. They serve to advocate for the issues most important to their constituencies. Between 1980 and 1999, Zimbabwe functioned as a de facto one-party state with very little opposition. The rise of the Movement of Democratic Change (MDC) in 1999 marked a shift in Zimbabwe’s democratic journey. For the last two decades, the MDC has enrichened political discourse and curtailed some of ZANU PFs worst tendencies. Mnangagwa’s regime is interested in closing this political space and creating a de facto one-party state.

In his words, Mr. Madhuku member of POLAD said, “POLAD is a useful platform that can be a way forward for Zimbabwe because it allows [political] losers to work with winners, and this must be adopted across African countries such as Zambia and Botswana. As NCA, we believe that it is an important way of governing the affairs of society, and voters need to know that governance is not only for those who won an election, but [also for] those that have lost so that the nation progresses by working together.” (Chifamba 2021a). Political analysts, including Alex Magaisa, Phelan Zamchiya, and Brian Raftopolous, interviewed for this paper, argue that POLAD is just another attempt to sow further division in the opposition and buy loyalty. Members of both POLAD and the PAC have been given a lot of perks, including luxury vehicles. In a country where the economy is failing, it is tough for individuals to walk away from such benefits. As a result, the country’s political opposition is more divided than it has ever been. Under such circumstances, it is easy for the ruling party to pit individuals against each other and bait weaker members with financial incentives. A weakened opposition has also made it harder for other civil society organizations to rally under a special message.

### Impact of ZANU PF’s authoritarianism on the civil society sector

Unsurprisingly, civil society activists have increasingly resorted to self-censorship and aligned their activities to fit the ZANU PF regime’s rules of engagement. Interviews with ten interest group leaders reveal that most people working within the CSO space are afraid to speak publicly. Nearly everyone interviewed asked that their identity be anonymized. This was true even for those talking positively about the government. It was also evident that some in civil society are now more interested in the development lenses, which aligns with the new government mandate. The government has claimed that only those organizations working toward education, food distribution, and other non-political activities can work openly. It is still too early to know if this promise is true.

Although most CSOs remain opposed to government interference, unlike in the Mugabe era, there appears to be a more significant schism in how CSOs respond to government restrictions. In an interview with the founder and leader of an organization that works on women’s and children’s rights, they said they had no issue with these new directives. They argued that their organization was compliant and that others should follow the law. When asked why they did not see these measures as problematic when they had previously lobbied against similar efforts during the Mugabe era, they shrugged and said, “things are different now. Mugabe is gone. We must rebuild the country” (Tariro, name changed, 2021). This is a common refrain from former opposition and civil society leaders who have opted to work with Mnangagwa. Another interviewee who worked for an organization dedicated to promoting voter participation shared the same sentiments adding that “we have to stop talking bad about our country.” (MARY, name changed, 2021).

One might say that there is some creeping fatigue with fighting losing battles against a government that will not likely lose power. For those who do not see a post-ZANU PF era anytime soon, it is much easier to cut their losses and work within the framework of the government. The same leader from the children and women’s organization also added that “not everything has to be political. I choose to focus on educating every child regardless of the party affiliation of their parents” (Tariro, name changed, 2021). The government has praised their organization as an example of good citizenship. It is too early to know if these organizations and individuals willing to tow the party line will continue to do so and if the government will treat them positively. It is also telling that although both Mary and Tariro had positive things to say about their experience working under the Mnangagwa regimes, they feared having their actual identities associated with their comments.

## Discussion and conclusion

The preceding discussion provides an insightful account of the nature of president Mnangagwa’s ZANU PF regime as a militarized former liberation movement whose singular focus is to consolidate and maintain political hegemony and control, which is incompatible with fostering an open democratic space in which civil society actors and opposition parties operate freely. Through the lens of the political settlements framework, we provide a comprehensive, granular analysis of the nature of the ZANU PF regime, the configurations of power in Zimbabwe’s politics, and the role that interest groups play in the country’s political dynamics. We make an important departure from the conventional view of civil society being fundamentally at odds with the authoritarian ZANU PF regime, arguing that the party uses the civil society an integral component of its strategy for maintaining power, to maintaining political legitimacy through the appearance of a democratic, pluralist government and national political system.

We argue that Mnangagwa’s government primarily seeks to maintain ZANU PF’s exclusive political settlement, which has been sustained since independence, entails entrenching political control via the regime’s primary sources of power: the armed forces, institutions of government, and revolutionary ideology. Interest groups, whose activities involve holding the government accountable, are at odds with the regime’s primary objectives, which involve maintaining access to the political and economic privileges that the exclusive settlement provides. However, since Mnangagwa and the ZANU PF party also seek to maintain a veneer of legitimacy by maintaining institutions and processes of a pluralist democratic system, which includes interest groups, this has led to a somewhat schizophrenic strategy of maintaining civil society groups’ existence while curtailing their activities to minimize their ability to threaten the regime’s grip on power and access to financial benefits.

This is exemplified by the desperate efforts of Mnangagwa’s government to distance itself from his predecessor, Robert Mugabe, by masquerading as a democrat when he came into power, initially opening the democratic space, allowing the return of political exiles, and establishing the PAC. Less than a year after Mnangagwa entered office, it became apparent that the nature of ZANU PF as a hegemonic, violent former military movement whose leaders are allergic to criticism, dissent, and accountability would be incompatible with an open civil society. Mnangagwa’s establishment of the POLAD and overtures to business leaders, churches, and private media houses are consistent with the ZANU PF ruling coalition’s strategies of divide and rule and carrot-and-stick approach to dealing with interest groups and the political opposition in general.

The co-option and the consequent fragmentation of some civil society organizations undermine their effectiveness as the Third Estate, whose mandate is to hold the government accountable. Mnangagwa’s overtures to the civil society seek to neutralize dissent by capitalizing on some civil society actors’ fatigue from fighting ZANU PF and seducing them with proximity to the power and patronage machinery of ZANU PF while posturing, for international audiences, as a democrat fostering an inclusive political space in Zimbabwe. The strategy of co-opting interest groups also signifies the crucial point that, despite establishing an exclusive political settlement, ZANU PF needs the civil society to exist to maintain the appearance of a democratic system, especially for foreign entities, especially multilateral donors such as the World Bank, and International Monetary Fund from whom the government aid, and foreign investors who would require a pluralistic political environment that, in theory, is more conducive for doing business.

As a result, independent interest groups and civil society organizations will continue to exist but operate within a restricted and periodically hostile political environment that ZANU PF’s exclusive political settlement engenders. Although some of the organizations may eventually succumb to their natural death either through actor fatigue, legislative authoritarianism, attack, or loss of financial resources, the ZANU PF government will unlikely pass legislation that completely bans civil society organizations but continually seek to constrain their activities to maintain its hegemonic control over the political system this does not bode well for the fate of democracy in Zimbabwe. Although the country has political institutions and the rituals of a well-functioning democracy, the incumbent ZANU PF party’s nature, interests, and sources of power remain incompatible with establishing a democratic regime that fosters culture and institutions of transparency, accountability, separation of powers, the rule of law, freedom of expression and association, and free and fair political competition that constitute the fabric of a well-functioning democratic society.

## Footnotes

1 ZANU PF’s organizational structure is modeled around Soviet-era political organization. The party’s Political Commissar is mainly responsible for defining and promoting the party’s political and ideological values and priorities and is thus one of the most influential positions one can hold in the organization.

2 See comment 2.

3 See comment 2.

4 Some of the most well-known businesspeople aligned with the ZANU PF regime include Kudakwashe Tagwirei, Billy Rautenbach, Nick Van Hoogstraten, and John Bredenkamp.

5 See, for instance Ken Yamamoto’s survey of corruption cases linked to ZANU PF, from 1980 to 2014 here: https://www.thezimbabwean.co/2016/05/robert-mugabes-corruption-1980-2014/

6 See, for instance Maier, K, https://www.washingtonpost.com/archive/politics/1989/04/15/3-cabinet-ministers-quit-in-zimbabwe-as-corruption-report-is-published/31e0f5ce-269d-49b3-832c-230129281606/

7 Herald Newspaper, 5 April 2004, comments attributed to Minister of Public Service, Labour and Social Welfare, Paul Mangwana.
